# High CO_2_ Reduces Spoilage Caused by *Botrytis cinerea* in Strawberry Without Impairing Fruit Quality

**DOI:** 10.3389/fpls.2022.842317

**Published:** 2022-04-27

**Authors:** Hua Li, Yuwei Yin, Fahrizal Yusuf Affandi, Caihong Zhong, Rob E. Schouten, Ernst J. Woltering

**Affiliations:** ^1^Horticulture and Product Physiology, Wageningen University and Research, Wageningen, Netherlands; ^2^Bioresource Technology and Veterinary Department, Vocational College, Universitas Gadjah Mada, Yogyakarta, Indonesia; ^3^Key Laboratory of Plant Germplasm Enhancement and Specialty Agriculture, Wuhan Botanical Garden, Chinese Academy of Sciences, Wuhan, China; ^4^Wageningen Food & Biobased Research, Wageningen, Netherlands

**Keywords:** *Fragaria × ananassa*, CA storage, shelf life, stepwise atmosphere, grey mold disease, pH, sugar and acid metabolism

## Abstract

High CO_2_ (> 20 kPa) conditions are beneficial for suppressing spoilage caused by *Botrytis cinerea* in strawberry fruit; however, these conditions are often accompanied by discoloration, off-flavors, and faster softening. Stepwise increments of CO_2_ concentrations have been proposed to alleviate injuries in fruits caused by high CO_2_. In this study, we investigated whether stepwise increments of CO_2_, up to 30 kPa and under a reduced O_2_ concentration, are beneficial for reducing fungal spoilage without inducing CO_2_ injury symptoms in strawberry fruit. Based on recommended settings (5–10 kPa O_2_ with 15–20 kPa CO_2_), we first selected optimal O_2_ and CO_2_ concentrations that best-reduced spoilage caused by *B. cinerea* in red ripe “Sonsation” strawberry fruit. We found that higher O_2_ (10 kPa) and CO_2_ (20 kPa) concentrations were most beneficial for prolonging strawberry fruit shelf life. Subsequently, we studied the performance of red ripe “Arabella” strawberry fruit stored at 5°C under different controlled atmosphere (CA) conditions (10 kPa O_2_ with either 0, 20, or 30 kPa CO_2_). The CO_2_ concentrations were achieved either within 8 h or in a stepwise manner within the first 4 days of storage. As a control, 21 kPa O_2_ and 0 kPa CO_2_ were used. Following storage for up to 11 days, the spoilage incidence was assessed at 12°C for 5 days. The application of high CO_2_ (20 and 30 kPa) combined with 10 kPa O_2_ greatly suppressed fruit spoilage during storage and subsequent shelf life. High CO_2_ suppressed respiration as well as maintained a higher pH and firmness in treated fruit. The level of total sugars did not change, but during storage, a substantial part of sucrose was converted into glucose and fructose, especially under high CO_2_ conditions. High CO_2_ did not affect ascorbic acid and anthocyanin levels. The stepwise increments of CO_2_ did not result in beneficial effects compared to the static application of high CO_2_. Our results show that “Arabella” strawberry fruit are highly tolerant to elevated CO_2_ and can be stored under 30 kPa CO_2_ to prolong the shelf life.

## Introduction

Strawberry fruit are highly appreciated due to their desired quality characteristics, such as “heart shape,” shiny skin, full red color, sweetness, distinct aroma, and abundant nutritional compounds (Madrid, 2020). However, high perishability limits the shelf life of strawberry fruit. Among pathogens, the ubiquitous fungi *Botrytis cinerea* caused the most losses in strawberry production (Petrasch et al., 2019), particularly in ripe fruit during postharvest storage. The strategy of combining low temperature (LT) and controlled atmosphere (CA, i.e., high CO_2_ and low O_2_ concentrations) has been widely used in the supply chain to retain product quality for market demand. For strawberry fruit, a postharvest condition of 5–10 kPa O_2_ and 15–20 kPa CO_2_ at 0–5°C is recommended to extend the storage period (Kader, 2003). The suggested concentrations of O_2_ (5–10 kPa) decreased respiration in strawberry fruit without inducing fermentation, thereby extending the storage life (Kanellis et al., 2009). Similarly, high CO_2_ concentrations between 15 and 20 kPa further suppressed respiration, delayed ripening, and senescence (Li et al., 2019) and may affect the acidity of strawberry fruit (Rama and Narasimham, 2003). In addition, high CO_2_ concentrations are fungistatic to *B. cinerea* since the fungus did not grow above 30 kPa CO_2_ (Garcia-Gimeno et al., 2002). However, high CO_2_ concentrations, above 20 kPa, may also accelerate strawberry softening and impair coloration, taste, and antioxidant levels (Wszelaki and Mitcham, 2000; Nakata and Izumi, 2020), thereby reducing fruit’s commercial value.

Antioxidants such as ascorbic acid and anthocyanins may play a role in protecting fruit from fungal infection. Ascorbic acid showed inhibitory effects on *B. cinerea* growth in apple fruit (Davey et al., 2007; Bui et al., 2019) and tomato fruit (Bassolino et al., 2013; Zhang et al., 2013). High CO_2_ concentrations may cause the oxidation of ascorbic acid or inhibit the reduction of dehydroascorbic acid. For instance, 10–30 kPa CO_2_ decreased ascorbic acid content in berry species, especially strawberry fruit (Agar et al., 1997; Shin et al., 2008). Similarly, the application of 20–40 kPa CO_2_ to “Selva” strawberry fruit reduced fungal spoilage but induced discoloration and anthocyanin reduction, which was presumably caused by an increase in pH or a decrease in co-pigmentation with flavonols and other phenolics (Gil et al., 1997; Nakata and Izumi, 2020).

Soluble sugars and non-voltile organic acids contribute to the sweetness and acidity of fruit (Schwieterman et al., 2014), and these compounds are also substrates for respiratory metabolism (Saltveit, 2019). Soluble sugar content and acidity of “Camarosa” strawberry fruit declined after a 3-day 20 kPa CO_2_ treatment when compared to fruit under ambient atmosphere (Bodelón et al., 2010). High CO_2_ concentrations may also lead to the production of fermentative volatile compounds. The application of ambient oxygen combined with 20 kPa CO_2_ to “Camarosa” strawberry fruit resulted in a shift in the synthesis of methyl-to-ethyl esters, contributing to off-flavors (Pelayo-Zaldívar et al., 2007).

A novel CA strategy has been proposed recently to extend blueberry shelf life: a stepwise increment of CO_2_ concentration at the start of CA storage (Falagán et al., 2020). A stepwise increment of CO_2_, reaching 10 kPa CO_2_ in 3 or 7 days (under 5 kPa O_2_), was applied. This resulted in a lower CO_2_ production peak, a reduced spoilage incidence, higher firmness, and higher ascorbic acid levels compared to static CA (5 kPa O_2_ and 10 kPa CO_2_) or ambient atmosphere conditions.

In this study, we aimed to investigate if stepwise increments of high CO_2_ extend strawberry shelf life without damaging important quality characteristics such as firmness, color, and nutrient content. We first determined the spoilage incidence by *B. cinerea* in strawberry fruit as a function of O_2_ and CO_2_ concentrations, which were based on recommended settings (Kader, 2003). High concentrations of both O_2_ (10 kPa) and CO_2_ (20 kPa) resulted in lower spoilage incidences and similar levels of firmness and sweetness compared to the other tested O_2_ and CO_2_ concentrations. In a second experiment, we investigated the effects of both static and stepwise increments of CO_2_ concentrations, up to 20 and 30 kPa, on the spoilage incidence, nutritional compounds, and antioxidant content during shelf life. The highest CO_2_ level, 30 kPa, considerably lowered the spoilage incidence and respiration rate without causing any nutrient or firmness loss in strawberry fruit. However, stepwise increments of CO_2_ concentrations did not provide any additional benefits compared to static CA storage at 20 or 30 kPa CO_2_.

## Materials and Methods

### Strawberry Fruit and Experimental Setup

#### Experiment I: Spoilage Evaluation Test Using Different CA Settings

Strawberry fruit (*Fragaria* × *ananassa* cv. Sonsation) were harvested in November 2019 from a greenhouse located in Dongen, Netherlands. Fruit were stored at 4°C overnight and were transported to the lab at Wageningen University and Research the next day (day 0). Red ripe fruit (with calyxes attached) without mechanical damage or visible spoilage were collected into round plastic cups (ø 10 cm × H 5 cm) and covered with a transparent lid. Each lid had nine small holes to allow sufficient gas diffusion. A total of 150 cups were packed, and the initial weight of all the cups with fruit was individually recorded. Six cups were used for chemical analyses on day 0. The remaining 144 cups were divided into cups of 24 and distributed over six CA treatments, resulting in three blocks per treatment. Six 70-L stainless steel CA containers were used. The CA containers were connected to a flow-through system flushing humidified gas mixture at a flow rate of 250 ml min^–1^ for the duration of the experiment. Strawberry fruit were subjected to the following CA treatments (balanced with N_2_ in all treatments):

(i) Static 10 O_2_ kPa + 10 CO_2_ kPa;
(ii) Static 5 O_2_ kPa + 15 CO_2_ kPa;
(iii) Static 7.5 O_2_ kPa + 15 CO_2_ kPa;
(iv) Static 10 O_2_ kPa + 15 CO_2_ kPa;
(v) Static 10 O_2_ kPa + 20 CO_2_ kPa;
(vi) Control: Static 20 O_2_ kPa + 0 CO_2_ kPa.

The set points for CA treatments were reached within 5–8 h. Fruit were treated for 0, 3, 6, 9, and 12 days under different CA conditions at 5°C and ∼100% relative humidity. Following CA storage, the fruit were held for 3 days under an ambient atmosphere at 12°C and ∼95% relative humidity. The CA containers were opened and closed within 2 min when strawberry fruit had to be measured for minimal disturbance of the atmosphere. Six cups per CA container per time point were sampled for measurements. Spoilage incidence, weight, firmness, and soluble solids content (Brix) were measured immediately after the CA treatments (3 cups per treatment) and after 3 days of shelf life at ambient temperature (3 cups per treatment).

#### Experiment II: Physiological Changes During Static and Stepwise Increments of CO_2_ Concentrations

Strawberry fruit (*Fragaria* × *ananassa* cv. Arabella) were grown in an open field tabletop system in Genderen, Netherlands, and harvested in August 2020. Fruit were cooled down for several hours before being transported to the lab at Wageningen University and Research (day 0). Red ripe fruit (with calyx still attached) without mechanical damage or visible spoilage were selected and collected into paper punnets with approximately 25 fruit per punnet. A total of 98 punnets were packed and individually weighed. Two punnets were used for chemical analyses on day 0. The remaining 96 punnets were divided into punnets of eight and distributed over six CA treatments, resulting in two blocks per treatment. Twelve 70-L stainless steel CA containers were used, and two for each treatment. The CA containers were connected to a flow-through system flushing humidified gas mixture at a flow rate of 250 ml min^–1^ for the duration of the experiment. Strawberry fruit were subjected to the following CA treatments (balanced with N_2_ in all treatments):

(i) GCA30: 10 kPa O_2_ with a stepwise increment to 30 kPa CO_2_ (7.5 kPa per day in 4 days);
(ii) GCA20: 10 kPa O_2_ with a stepwise increment to 20 kPa CO_2_ (5 kPa per day in 4 days);
(iii) CA30: Static10 kPa O_2_ + 30 kPa CO_2_;
(iv) CA20: Static10 kPa O_2_ + 20 kPa CO_2_;
(v) CA0: Static 10 kPa O_2_ + 0 kPa CO_2_;
(vi) Control: Static 20 O_2_ kPa + 0 CO_2_ kPa.

The set points for all CA treatments were reached within 5–8 h. Fruit were stored for 11 days under different CA conditions at 5°C and ∼100% relative humidity; thereafter, they were held for 5 days under an ambient atmosphere of 12°C and ∼95% relative humidity (shelf life). The CA containers were opened and closed within 2 min when strawberry fruit were removed for measurements. One individual CA unit (regarded as one replication) contained 8 punnets as described above. During the CA treatment, fruit were sampled on days 4 and 11; during the subsequent shelf life, fruit were sampled on day 3 (total period 14 days) and day 5 (total period 16 days). At each sampling point, one punnet (approximately 25 fruit) per treatment was randomly taken for quality analyses, including fresh weight, firmness, and biochemical compounds. Another 4 punnets (approximately 100 fruit) were used for non-destructive spoilage incidence evaluation on day 11 of CA storage and days 3 and 5 of shelf life.

Both “Sonsation” and “Arabella” strawberry fruit are commercially used with good firmness, medium red color, good tolerance to *B. cinerea*, and prolonged shelf life during storage. “Sonsation” is a short-day cultivar, whereas “Arabella” has the potential to crop over an extended season. Since “Sonsation” fruit were no longer available in July 2020, we chose a very similar cultivar for Experiment II.

### Respiration Rate

In Experiment II, the respiration rate of the fruit under different CA conditions was monitored. For this, the outlet of each CA container was connected to a 2 L glass cuvette filled with approximately 600–750 g of fruit. In this way, the cuvettes received the same gaseous conditions as the other stored fruit. At the start of the respiration measurement, the glass cuvettes were disconnected from the flow-through system and all valves were closed. Concentrations of O_2_ and CO_2_ were measured immediately after closure and again after about 6 h of incubation; from this, the respiration rate was calculated. The calculation considered the O_2_ consumption rate, cuvette volume, and fruit mass; the results were expressed in nmol O_2_ kg^–1^ s^–1^. CO_2_ and O_2_ were measured using a headspace gas analyzer (Checkmate 3, PBI Dansensor, Ringstead, Denmark).

### Spoilage Incidence and Weight Loss

Fruit showed typical *B. cinerea* infection symptoms (e.g., brown spots or visible gray mycelium). Only fruit showing Botrytis rot symptoms (Feliziani and Romanazzi, 2016; Petrasch et al., 2019) were considered for spoilage calculation. Botrytis rot was the pivotal fungal pathogen throughout the experiment. Occasionally, some other fungal symptoms were observed, such as Rhizopus fruit rot, and these were not considered in the calculation. The spoilage incidence was expressed as the percentage of fruit per block affected by a fungal infection. The weight of an individual cup or punnet with fruit was recorded using an MS6002TS balance (Mettler-Toledo GmbH, Giessen, Germany) at harvest. Weight loss was expressed as the percentage of weight loss compared to the initial fruit weight, corrected for the weight of cups or punnets.

### Firmness and Brix

In Experiment I, firmness (limited compression) was measured using FirmTech FT7 (UP GmbH, Ibbenbüren, Germany). Firmness was expressed as the force-displacement (in g mm^–1^) after about 1 mm of compression of the fruit shoulder without a calyx. Each replicate (cup) had 5 fruit. The same fruit were used for Brix measurement using a refractometer (PAL-1, Atago, Japan).

In Experiment II, fruit firmness (penetration) was measured using a universal testing machine (Zwick Z2.5/TS1S materials testing machine; Ulm, Germany). Fruit were cut in half lengthwise and compressed at the equator with a probe (ø 2.5 mm) at a constant plunger speed (2.5 mm s^–1^) to a fixed penetration depth (5 mm). The maximum force (N) was recorded during the penetration of the fruit piece. Each replicate (punnet) had 15 strawberry fruit.

### Dry Weight Percentage and pH

Fruit from one punnet (approximately 25 fruit) were cut into small pieces, immediately frozen in liquid N_2_, and ground into powder. Part of the frozen powder was freeze-dried for sugar and organic acid extraction and also for expressing all nutrients as per gram dry weight. Frozen powder (0.3 g) was dissolved in 1.5 ml of water, thoroughly shaken, and centrifuged at 21,000 × *g* at 20°C for 15 min. The supernatant was used for pH measurement.

### Ascorbic Acid

Frozen powder (0.2 g) was thawed on ice in darkness with 1 ml of ice-cold 3.3% meta-phosphoric acid. Samples were sonicated for 10 min, then centrifuged at 21,000 × *g* at 4°C for 10 min. The supernatant was filtered through a 0.45 μm cellulose filter and injected into an HPLC system consisting of a GS50 pump (Dionex), a 340S UV-VIS detector (Dionex), and a MIDAS autosampler (Spark Holland) equipped with a ProntoSIL 120-3 C18 AQ, 250 mm × 3 mm column (Knauer). The column was eluted with 400 μl L^–1^ H_3_PO_4_ + 2.5 ml L^–1^ MeOH + 0.1 mM EDTA in distilled water, followed by a wash step with 30% acetonitrile in distilled water at a flow rate of 0.35 ml min^–1^ at 35°C. Ascorbic acid was detected at 243 nm. The system was calibrated using an authentic ascorbic acid standard (Acros Organics) prepared at 3.3% MPA.

### Anthocyanins

The frozen sample (0.3 g) was mixed with 1.5 ml of 50% methanol containing 1% formic acid extraction solvent. The supernatant was filtered through a 0.45 μm cellulose filter prior to injection into an HPLC system (Ultimate 3000, Dionex, Sunnyvale, CA, United States). Solvents used were (A) 0.1% trifluoroacetic acid in distilled water, and (B) 0.1% trifluoroacetic acid in HPLC-grade acetonitrile, establishing the following gradient: 5–28% of B for 0–35 min, 28–75% of B for 35–37 min, isocratic 75% of B for 37–40 min, 75–5% of B for 40–42 min, and isocratic 5% of B for 42–50 min, using a flow rate of 0.8 ml min^–1^. Separation was achieved using a C18 column (150 mm × 3 mm, 3 μm; HyPURITY C18, Thermo Scientific, New York, NY, United States), and peaks were identified using a UV/vis detector at 520 nm using pelargonidin 3-glucoside as an authentic standard (Extrasynthese).

### Sugars, Citric Acid, and Malic Acid

A total of 15 mg of freeze-dried tissue was mixed with 5 ml of 75% ethanol, followed by shaking and incubating in a water bath at 80°C for 20 min before centrifuging at 8,500 × *g* for 5 min at 4°C. A vacuum centrifuge (Savant SpeedVac SPD2010, Thermo Fisher Scientific, Germany) was used to dry 1 ml of the supernatant at 55°C for 2.5 h. The pellet was resuspended in 1 ml of distilled water and sonicated for 10 min, followed by centrifugation for 10 min at 4°C at 14,800 × *g*. Before analysis, the supernatant was diluted with distilled water 50 times for sugar and 5 times for organic acids.

Soluble sugar measurements were carried out using a High-Performance Anion Exchange Chromatography with Pulsed Amperometric Detection (HPAEC-PAD; Dionex ICS5000, Thermo Fisher Scientific, Germany) equipped with a CarboPac1 column (250 mm × 2 mm) eluted with 100 mM NaOH at a flow rate of 0.25 ml min^–1^ at 25°C. Quantification was performed using glucose, fructose, and sucrose standards from Sigma-Aldrich.

Citric and malic acids in the extracts were analyzed using an IC system equipped with a Triathlon autosampler (Spark Holland), GS50 pump (Dionex), ED50A detector (Dionex) operating in the conductivity mode, and an ASRS ultra II 2 mm suppressor (Dionex). Anions were separated at 30°C on an IonPac AS11HC (250 mm × 2 mm) column (Dionex), using the following multistep gradients: 1 mM NaOH, 0 min; 1 mM NaOH, 1 min; 14 mM NaOH, 23 min; 30 mM NaOH, 31 min; 60 mM NaOH, 41 min at a flow rate of 0.38 ml min^–1^. Quantification was performed using authentic DL-malic acid and citric acid standards from Sigma-Aldrich.

### Statistical Analysis

Treatment effects on all measured variables were tested using a one-way analysis of variance (ANOVA) at each time point during the experimental period. Experiment I was carried out with 3 blocks, and Experiment II was carried out with 2 blocks. In each block, replicated fruit were measured as an individual or a pooled sample, depending on variables. An average value of each block was used for statistical analysis. Homogeneity and normality of residuals in the ANOVA were tested using Bartlett’s test and Shapiro–Wilk test, respectively. Fisher’s protected least significant difference (LSD) test was used as a *post hoc* test. All statistical analyses were performed in Genstat (19th edition, VSN International Ltd., Hemel Hempstead, United Kingdom). All tests were conducted at α = 0.05.

## Results

### Spoilage Incidence of “Sonsation” Strawberry Fruit

**FIGURE 1:**
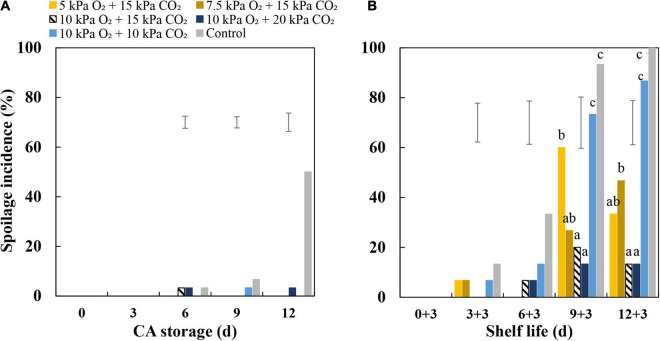
Spoilage incidence of “Sonsation” strawberry fruit stored under static controlled atmosphere (CA) conditions and shelf life. **(A)** Spoilage incidence at different time points during CA storage at 5°C and ∼100% relative humidity. **(B)** Spoilage incidence at different time points of CA storage followed by 3-day shelf life at 12°C and ∼95% relative humidity in ambient atmosphere. The first number is the number of days in CA storage, and the second number is the number of days in shelf life. As a control condition, 21 kPa O_2_ and 0 kPa CO_2_ were used. Data represent means of 3 blocks (*n* = 3) with five replicated fruit per block. The error bars represent the standard error of means. Different letters denote significant differences according to Fisher’s protected LSD test (α = 0.05).

During CA storage, the spoilage incidence of CA-treated fruit was lower compared to control-treated fruit (Figure 1A). During shelf life, the spoilage incidence increased dramatically in fruit from the ambient atmosphere and 10 kPa CO_2_ + 10 kPa O_2_ (Figure 1B). Fruit stored at 15 kPa CO_2_ showed reduced spoilage incidence with increasing O_2_ concentrations from 5 to 10 kPa. Under conditions of 10 kPa O_2_, fruit treated with 15 and 20 kPa CO_2_ showed the lowest spoilage incidence among all treatments. Overall Brix values of fruit showed a decreasing trend during CA storage but did not show a further change during the 3-day subsequent shelf life; fruit firmness did not change during CA storage but showed a slight decrease during the 3-day subsequent shelf-life period. The different treatments had no significant effects on the changes of Brix values and the firmness of fruit (Supplementary Figure 1).

### Respiration Rate and Spoilage Incidence of “Arabella” Strawberry Fruit

**FIGURE 2:**
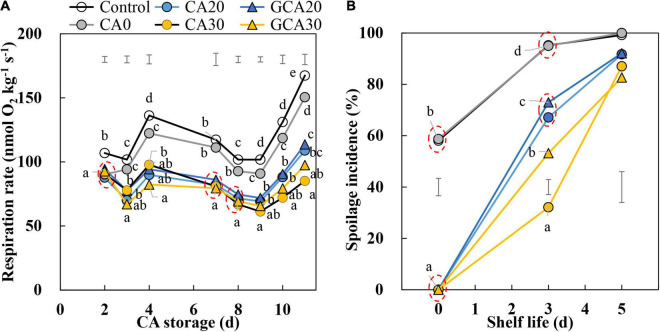
Respiration rate and spoilage incidence of “Arabella” strawberry fruit during CA storage and shelf life. **(A)** Respiration rate during CA storage at 5°C and ∼100% relative humidity. **(B)** Spoilage incidence during shelf life at 12°C and ∼95% relative humidity in ambient atmosphere. As a control condition, 21 kPa O_2_ and 0 kPa CO_2_ were used. Data represent means of 2 blocks (*n* = 2) with approximately 35 replicated fruit for respiration and approximately 25 replicated fruit for spoilage measurements per block. The error bars represent the standard error of means. Different letters denote significant differences according to Fisher’s protected LSD test (α = 0.05).

The fruit respiration rate was higher in CA0 and the control conditions compared to the elevated CO_2_ treatments (20 and 30 kPa), indicating that increased CO_2_ concentrations suppressed respiration (Figure 2A). The respiration rate fluctuated during the first 5 days of storage, regardless of treatments. After day 9, the respiration rate started to increase. This increase was smaller and not significant for fruit stored under 30 kPa CO_2_ conditions. There were no apparent differences in respiration related to the immediate or stepwise application of high CO_2_ (20 and 30 kPa).

Fruit stored under CA0 and control conditions showed high spoilage incidence at the start of shelf life (Figure 2B). The spoilage incidence of CA0 overlapped with that of the control treatment, reaching about 58% spoilage at the end of the CA storage. In contrast, fruit stored under elevated CO_2_ with 10 kPa O_2_ treatments did not show any spoilage during the CA storage; during the subsequent shelf life at 12°C and ambient atmosphere, fruit spoilage did occur in these treatments. After the 3-day shelf life, fruit stored under CA30 had the lowest spoilage incidence (32%), followed by GCA30 (53%) and CA20 and GCA20 (over 60%). After 5 days of shelf life, the spoilage incidence was above 80% in all treatments.

### Sugar Levels of “Arabella” Strawberry Fruit

**FIGURE 3:**
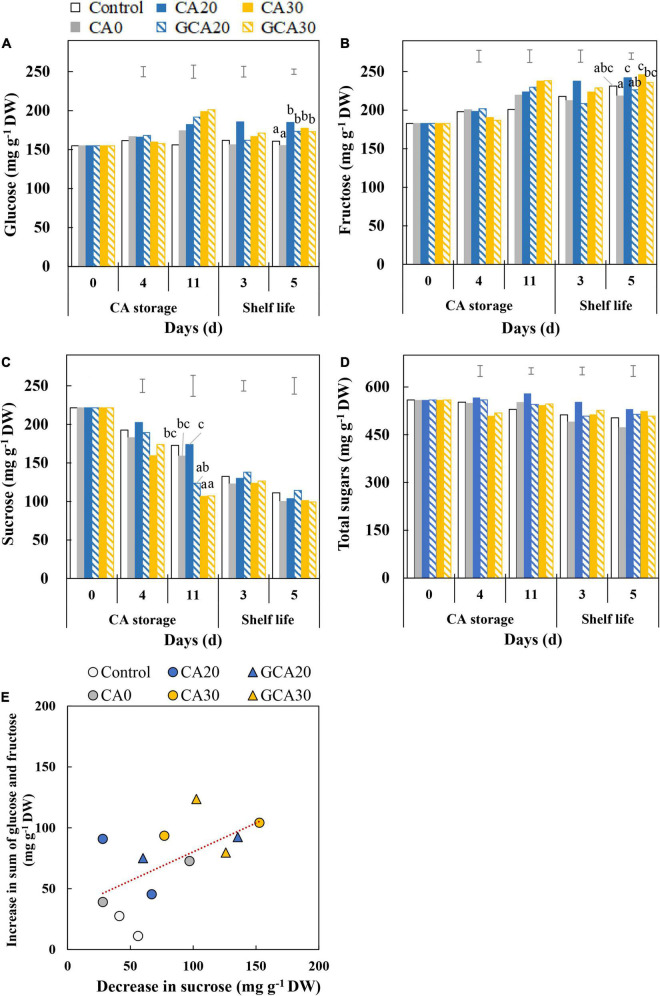
Soluble sugars of “Arabella” strawberry fruit during CA storage and shelf life. **(A)** Glucose, **(B)** fructose, **(C)** sucrose, and **(D)** total sugar (sum of glucose, fructose, and sucrose) during CA storage at 5°C and ∼100% relative humidity and subsequent shelf life at 12°C and ∼95% relative humidity in ambient atmosphere. Data represent means of 2 blocks (*n* = 2; each block being a pooled sample of 20 replicated fruit). Different letters denote significant differences according to Fisher’s protected LSD test (α = 0.05). The error bars represent the standard error of means. **(E)** Correlation between the increased content of glucose and fructose and the decreased content of sucrose between day 0 and day 11 during CA storage. Data represent individual blocks. As a control condition, 21 kPa O_2_ and 0 kPa CO_2_ were used.

During the CA storage and shelf life, the glucose content did not show a clear trend (Figure 3A), whereas the fructose content increased (Figure 3B), and the sucrose content decreased (Figure 3C). The total sugar content (glucose + fructose + sucrose) showed a downward trend for all treatments (Figure 3D). No apparent differences in the rate of change in individual sugars among treatments were observed. However, the sucrose content in fruit from both the static and stepwise 30 kPa CO_2_ treatments showed a more rapid decrease in the CA storage than the sucrose level in fruit from other treatments (Figure 3E). This indicates that under high CO_2_ conditions, there was a more pronounced conversion of sucrose into glucose and fructose.

### pH and Organic Acids of “Arabella” Strawberry Fruit

**FIGURE 4:**
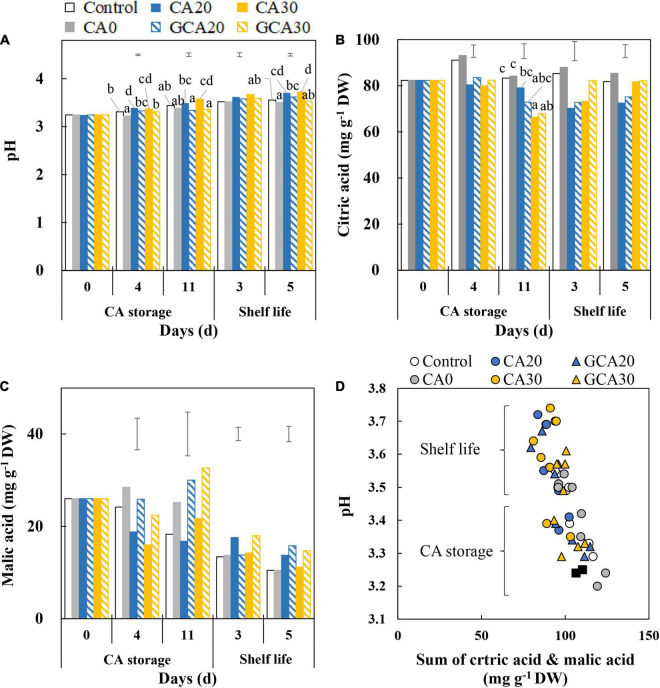
pH and main organic acids of “Arabella” strawberry fruit during CA storage and shelf life. **(A)** pH, **(B)** citric acid, and **(C)** malic acid during CA storage at 5°C and ∼100% relative humidity and subsequent shelf life at 12°C and ∼95% relative humidity in ambient atmosphere. Data represent means of 2 blocks (*n* = 2; each block being a pooled sample of 20 replicated fruit). The error bars represent the standard error of means. Different letters denote significant differences according to Fisher’s protected LSD test (α = 0.05). **(D)** Correlation between pH and sum of citric and malic acids of CA treatments during CA storage and subsequent shelf life. The closed black squares represent data of day 0. Data represent individual blocks. As a control condition, 21 kPa O_2_ and 0 kPa CO_2_ were used.

Fruit pH increased from about 3.2 to 3.7 with minimal differences between treatments (Figure 4A). Fruit from the CA30 condition generally showed a slightly higher pH than the control and CA0 treatments. Citric acid did not show any changes throughout the CA storage and shelf life, and no treatment effects were observed (Figure 4B). The malic acid content in fruit was 2.2-fold lower than citric acid at harvest and showed a slight decrease after fruit were transferred to the shelf life condition in all treatments. No differences with respect to the CA treatments were observed (Figure 4C). The increase in pH during the experimental period can be ascribed to the decrease in the two major acids; fruit pH during the CA storage was lower than during the subsequent shelf life, regardless of atmospheric composition. In addition, high CO_2_ treatments led to a higher pH compared to control and CA0 treatments after fruit were transferred to the ambient atmosphere (Figure 4D).

### Firmness and Weight Loss of “Arabella” Strawberry Fruit

**FIGURE 5:**
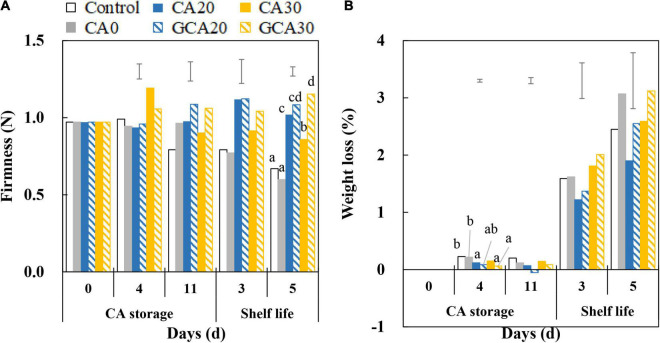
Firmness and weight loss of “Arabella” strawberry fruit during CA storage and shelf life. **(A)** Firmness and **(B)** weight loss during CA storage at 5°C and ∼100% relative humidity and subsequent shelf life at 12°C and ∼95% relative humidity in ambient atmosphere. Data represent means of 2 blocks (*n* = 2; each block being averaged of 20 replicated fruit). The error bars represent the standard error of means. Different letters denote significant differences according to Fisher’s protected LSD test (α = 0.05). As a control condition, 21 kPa O_2_ and 0 kPa CO_2_ were used.

Softening was observed in fruit from control and CA0 treatments during the subsequent shelf life (Figure 5A). Fruit from high CO_2_ treatments (except for CA30) retained their firmness during the shelf life and no promising effect of the stepwise elevation of CO_2_ was observed. Fruit weight loss during the CA storage was minimal; in fact, fruit weight loss increased up to a maximum of 3% in the subsequent shelf life, and no differences were apparent between treatments (Figure 5B).

### Ascorbic Acid and Anthocyanin Levels of “Arabella’ Strawberry Fruit

**FIGURE 6:**
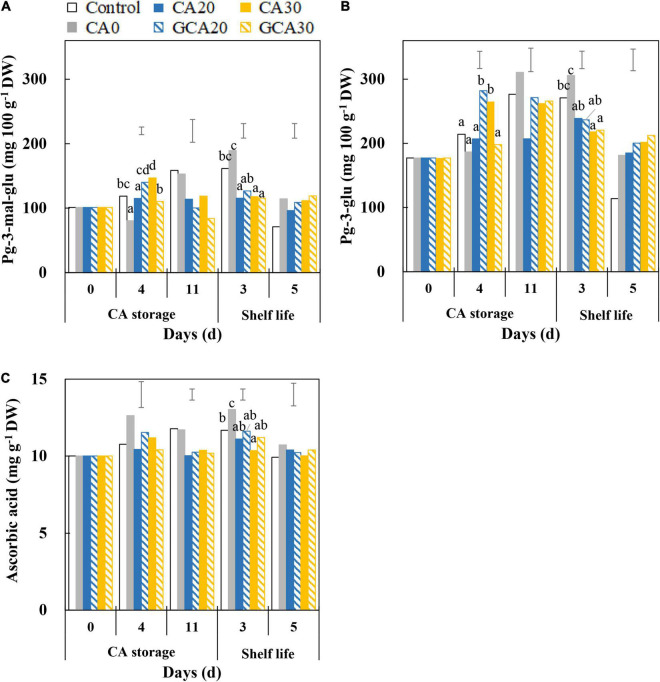
Anthocyanin and ascorbic acid content of “Arabella” strawberry fruit during CA storage and shelf life. **(A)** Pelargonidin-3-malonyl glucoside, **(B)** pelargonidin-3-glucoside, and **(C)** ascorbic acid during CA storage at 5°C and ∼100% relative humidity and subsequent shelf life at 12°C and ∼95% relative humidity in ambient atmosphere. As a control condition, 21 kPa O_2_ and 0 kPa CO_2_ were used. Data represent means of 2 blocks (*n* = 2; each block being a pooled sample of 20 replicated fruit). The error bars represent the standard error of means. Different letters denote significant differences according to Fisher’s protected LSD test (α = 0.05).

Pelargonidin-3-glucoside and pelargonidin-3-malonyl glucoside were the major anthocyanins in “Arabella” strawberry fruit, and the content of the former was lower (∼75%) than the content of the latter at harvest (Figures 6A,B). Overall, pelargonidin-3-glucoside content in fruit increased during the CA storage with decreasing trends during shelf life. There were no consistent differences between treatments. Similar trends were observed in the pelargonidin-3-malonyl glucoside content of fruit. Ascorbic acid levels of “Arabella” fruit showed slightly increasing trends in fruit from control and CA0 treatments throughout CA storage and shelf life. In contrast, the ascorbic acid level did not change in fruit treated with elevated CO_2_ concentrations, where it was maintained around 10 mg g^–1^ DW (Figure 6C). Collectively, until the end of shelf life, the antioxidants of fruit showed similar levels compared to fruit at harvest.

## Discussion

### Reduced O_2_ and High CO_2_ Are Simultaneously Required for Retarding Fungal Spoilage of Strawberry Fruit

Controlled atmosphere storage retains product quality *via* reducing O_2_ and increasing CO_2_ concentrations relative to the ambient atmosphere. Generally, the lower the O_2_ and the higher the CO_2_ concentrations, the more the storage life can be extended by delaying senescence and ripening of commodities (Rama and Narasimham, 2003). However, based on our observations, this concept may not fully apply to strawberry fruit. In Experiment I, moderately decreased O_2_ concentrations (5–10 kPa) combined with 15 kPa CO_2_ greatly decreased the spoilage incidence of “Sonsation” strawberry fruit, which was observed only after fruit were transferred to post-storage shelf life (Figure 1B). Notably, 10 kPa O_2_ clearly performed better than 5 or 7.5 kPa O_2_. Generally, when high CO_2_ is applied to strawberry fruit, the risk of initiating fermentation increases, which leads to the accumulation of acetaldehyde and ethanol, resulting in off-flavors (Rama and Narasimham, 2003; Kanellis et al., 2009). This could be prevented and/or attenuated by introducing more O_2_ into the environment. In contrast, Ke et al. (1991) found that decreasing concentrations of O_2_ (down to 0.25% O_2_ balanced with N_2_ only) led to reduced spoilage incidence in “Selva” strawberry fruit at both 0 and 5°C and did not affect the soluble solids content (brix), pH, or titratable acidity after 10-day CA treatments compared to air-treated fruit. However, this study also reported a higher ethanol content of fruit under this treatment compared to fruit from other treatments. It is unclear if these beneficial effects of ultralow O_2_ persisted during the shelf life, as authors only did quality measurements at the end of the storage period.

We observed that increased CO_2_ concentrations (10–20 kPa) combined with 10 kPa O_2_ drastically lowered the spoilage incidence of fruit. Fruit firmness and Brix did not exhibit differences during CA storage and shelf life among treatments (Supplementary Figure 1). Application of 20 kPa CO_2_ in air did not induce the fermentation of “Selva” strawberry fruit and ultimately resulted in a lower spoilage incidence (Ke et al., 1991). This shows that in order to benefit from CA storage, optimal O_2_ concentrations might not be lower than 10 kPa and CO_2_ should not be lower than 15 kPa.

Therefore, in Experiment II, we referred to the optimal 10 kPa O_2_ CA conditions for “Sonsation” strawberry fruit, which showed a lower spoilage incidence without affecting fruit firmness and Brix, and applied that in combination with different concentrations of CO_2_ to “Arabella” strawberry fruit. Both conditions with 10 kPa O_2_ and high (20 and 30 kPa) CO_2_ significantly suppressed spoilage of “Arabella” strawberry fruit. This confirms the results with “Sonsation” fruit, indicating the two cultivars may respond to high CO_2_ conditions similarly.

Notably, in Experiment II, the condition of 10 kPa O_2_ alone (Figure 2B, CA0) did not suppress the spoilage development compared to the control condition at the end of the CA storage. Combing the results of two experiments suggests that high CO_2_ combined with reduced O_2_ could be effective for reducing spoilage. When high CO_2_ is applied to strawberry fruit, the concentration of O_2_ should not be too low; otherwise, the additive effects of high CO_2_ and low O_2_ may induce fermentation.

### High CO_2_ Affected Sugar and Acid Metabolism

CO_2_ concentrations higher than 30 kPa can cause faster softening, berry discoloration, and the production of off-flavors, leading to quality loss. Elevating CO_2_ concentrations in a stepwise manner may improve the adaption of products to high CO_2_ compared to static CO_2_ treatments such that the storage life, fruit firmness, color, nutritional compounds, and antioxidants are retained (Falagán et al., 2020). Raising gas concentrations over a 3- or 7-day period to reach final concentrations of 10 kPa CO_2_ and 5 kPa O_2_ resulted in a lower CO_2_ production peak and reduced spoilage incidence in blueberries when compared to static CA or ambient atmospheric conditions (Falagán et al., 2020).

In this study, the respiration rate (Experiment II) of fruit under high CO_2_ treatments was reduced throughout the storage period compared to fruit from low CO_2_ treatments (Figure 2A). However, the pattern was not affected by applying high CO_2_ in a stepwise manner. Similarly, the spoilage incidence was greatly suppressed by high CO_2_ but was not affected by the stepwise application. The suppressed respiration caused by high CO_2_ is due to the inhibition of succinate dehydrogenase that catalyzes the conversion of succinate to fumarate at the tricarboxylic acid (Kanellis et al., 2009). Malic acid, as the upstream metabolite of succinate and fumarate, was reduced by high CO_2_ (20 kPa CO_2_ in air) (Fernández-Trujillo et al., 1999; Ponce-Valadez et al., 2005). The observed decreasing trend of malic acid, especially in fruit treated with elevated CO_2_ (Figure 4C), is in line with previous findings.

In strawberry fruit, sucrose is degraded *via* the invertase pathway to form fructose and glucose (Kanellis et al., 2009; Durán-Soria et al., 2020). We found that fructose and glucose in strawberry fruit slightly increased during the experimental period in all CA treatments, whereas sucrose decreased. High CO_2_ treatments seemed to induce a more significant conversion from sucrose to glucose and, in particular, fructose (Figure 3E). Bang et al. (2019) also observed increases in glucose and fructose but a decrease in sucrose content in strawberry fruit after 1 day of treatment with 30 kPa CO_2_ at 25°C compared to the ambient atmosphere condition. The transcriptome analysis indicated that the invertase inhibitor was downregulated under 30 kPa CO_2_, triggering invertase activity; therefore, more glucose and fructose are synthesized in fruit.

### High CO_2_ Did Not Impair Strawberry Firmness and Antioxidants or Induce Fermentation

A decreasing trend in firmness in control and CA0-treated fruit was observed, especially after the fruit were returned to the ambient atmosphere (Figure 5A). The firmness of fruit from high CO_2_ treatments was maintained at approximately the same value at the end of shelf life compared to the firmness at harvest. The different firmness behaviors between 0 kPa CO_2_ and high CO_2_ corroborate that CO_2_ treatments inhibit firmness loss of strawberry fruit in a non-reversible manner (Harker et al., 2000). Fruit treated with static 30 kPa CO_2_ tended to be softer compared to fruit from other high CO_2_ treatments. This could be due to the adverse effect of long-term storage under static 30 kPa CO_2_ as it was not observed under stepwise high CO_2_ treatments.

Increased firmness in strawberry fruit under elevated CO_2_ is likely due to reinforcement of cell-to-cell bonding, which is associated with the increase in pH of the apoplast (Harker et al., 2000). According to our finding, the pH of “Arabella” strawberry fruit increased throughout the whole experimental period due to a reduction in organic acids, especially malic acid (Supplementary Figure 2), which is in line with previous findings (Ke et al., 1991; Holcroft and Kader, 1999; Blanch et al., 2015). Both H^+^ and HCO_3_^–^ are produced by the solubilization of CO_2_, which could affect pH (Lucas, 1979; Bown, 1985). The presence of H^+^ decreased pH, whereas the uptake of HCO_3_^–^ into cells increased apoplastic pH due to the presence of an OH^–^ efflux instead of an H^+^ influx transport system (Laing and Browse, 1985). This increase in pH of the apoplast possibly enables Ca^2+^, rather than H^+^, as the ion species that binds to negatively charged carboxyl groups of the cell wall (Harker et al., 2000) to promote linking of neighboring pectin polymers through the egg-box model (Demarty et al., 1984). This idea is in line with our observations. We observed that fruit firmness increased during high CO_2_ treatment, whereas it slightly decreased after fruit were transferred to the ambient atmosphere (Figure 5A). In contrast, firmness continuously declined in fruit in the control and CA0 treatments.

The differences in the anthocyanin content of fruit from different treatments during CA storage and shelf life were not consistent, presumably due to biological variation in samples. The ascorbic acid content in fruit did not change over the experiment, and no differences between the control and different CA treatments were apparent. From these aspects, our findings in “Arabella” strawberry fruit are different from most studies which found firmness loss, discoloration, and antioxidant losses in strawberry fruit treated with elevated CO_2_ (Gil et al., 1997; Lee and Kader, 2000; Shin et al., 2008; Li et al., 2019).

Application of high CO_2_ might induce fermentation. The extent of fermentation with increased CO_2_ concentrations is cultivar-dependent in strawberry fruit, and some cultivars do not produce fermentation metabolites at all (Fernández-Trujillo et al., 1999; Watkins et al., 1999; Pelayo-Zaldívar et al., 2007). As the ratio of CO_2_ production and O_2_ consumption in fruit approached 1, both under 20 and 30 kPa CO_2_ (data not shown), we assume that fermentative metabolism was not activated during CA storage. Therefore, “Arabella” strawberry fruit seem relatively tolerant to elevated CO_2_. This could explain that although high CO_2_ lowered the spoilage incidence, other quality properties, such as firmness, anthocyanins, and ascorbic acid, were hardly affected. The lower spoilage incidence not only resulted from the physiological effects of high CO_2_, as we discussed above but may also be due to the direct suppression of high CO_2_ on *B. cinerea* growth. *In vitro* inhibition tests showed that the *B. cinerea* colony diameter decreased with elevated CO_2_ from 0 to 20 kPa, and it could not grow when CO_2_ concentrations were higher than 30 kPa (Garcia-Gimeno et al., 2002). After fruit were transferred to the ambient atmosphere condition, the inhibitory effect was relieved, but the residual effect of elevated CO_2_ still existed, which was observed in both experiments and the two cultivars studied in our research.

## Conclusion

The application of high CO_2_ (20 and 30 kPa) combined with 10 kPa O_2_ effectively reduced strawberry fruit spoilage by *B. cinerea*, probably resulting from lower respiration rates and higher pH. High CO_2_ did not affect total soluble sugar content but caused a more pronounced conversion of sucrose to glucose and fructose. High CO_2_ did not induce fermentation or affect antioxidant levels of fruit. Stepwise increments of CO_2_ did not show beneficial effects on overall fruit quality compared to the static application of high CO_2_. “Arabella” strawberry fruit can be stored under 30 kPa CO_2_ for prolonged periods without loss of overall quality.

## Data Availability Statement

The original contributions presented in the study are included in the article/Supplementary Material, further inquiries can be directed to the corresponding author.

## Author Contributions

HL, CZ, and EW conceived and designed the experiments. HL and CZ conducted Experiment I. HL and YY conducted Experiment II and analyzed the data. HL wrote the manuscript with the help of EW. RS and FA provided critical comments on the overall structure of the manuscript. All authors reviewed and approved the final manuscript.

## Conflict of Interest

The authors declare that the research was conducted in the absence of any commercial or financial relationships that could be construed as a potential conflict of interest.

## Publisher’s Note

All claims expressed in this article are solely those of the authors and do not necessarily represent those of their affiliated organizations, or those of the publisher, the editors and the reviewers. Any product that may be evaluated in this article, or claim that may be made by its manufacturer, is not guaranteed or endorsed by the publisher.

## Supplementary Material

Supplementary Figure 1 Brix and firmness of “Sonsation” strawberry fruit stored under controlled atmosphere (CA) conditions and shelf life. **(A)** Brix and **(C)** firmness at different time points during CA storage at 5°C and 100% relative humidity. **(B)** Brix and **(D)** firmness at different time points of CA storage followed by 3-day shelf life at 12°C and 100% relative humidity in ambient atmosphere. As a control condition, 21 kPa O_2_ and 0 kPa CO_2_ were used. Data represent means of 3 blocks (*n* = 3) with five replicate fruit per block. The error bars represent the standard error of means. Different letters denote significant differences according to Fisher’s protected LSD test (α = 0.05).

Supplementary Figure 2 Correlation between pH and firmness of fruit from different CA treatments during storage and subsequent shelf life. The closed black squares represent data of day 0. Data represent individual blocks.
