# Supplementary figures and images for: High CO2 Reduces Spoilage Caused by Botrytis cinerea in Strawberry Without Impairing Fruit Quality
Source: Front Plant Sci. 2022 Apr 27;13:842317. doi: 10.3389/fpls.2022.842317 (PMC9094085; doi:10.3389/fpls.2022.842317)

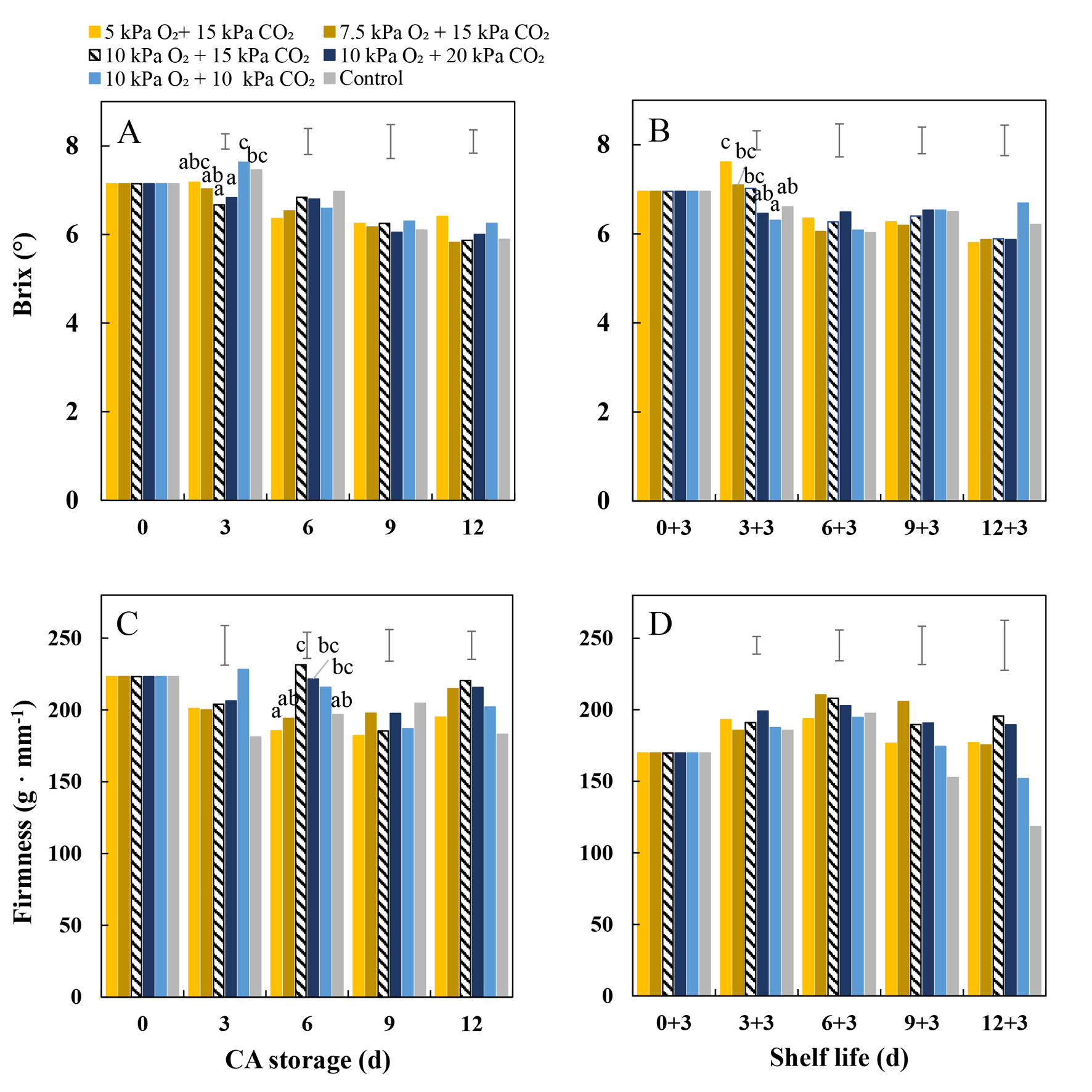

Supplement: Supplementary Figure 1 — Brix and firmness of “Sonsation” strawberry fruit stored under controlled atmosphere (CA) conditions and shelf life. (A) Brix and (C) firmness at different time points during CA storage at 5°C and 100% relative humidity. (B) Brix and (D) firmness at different time points of CA storage followed by 3-day shelf life at 12°C and 100% relative humidity in ambient atmosphere. As a control condition, 21 kPa O2 and 0 kPa CO2 were used. Data represent means of 3 blocks (n = 3) with five replicate fruit per block. The error bars represent the standard error of means. Different letters denote significant differences according to Fisher’s protected LSD test (α = 0.05). [file Image_1.TIF]

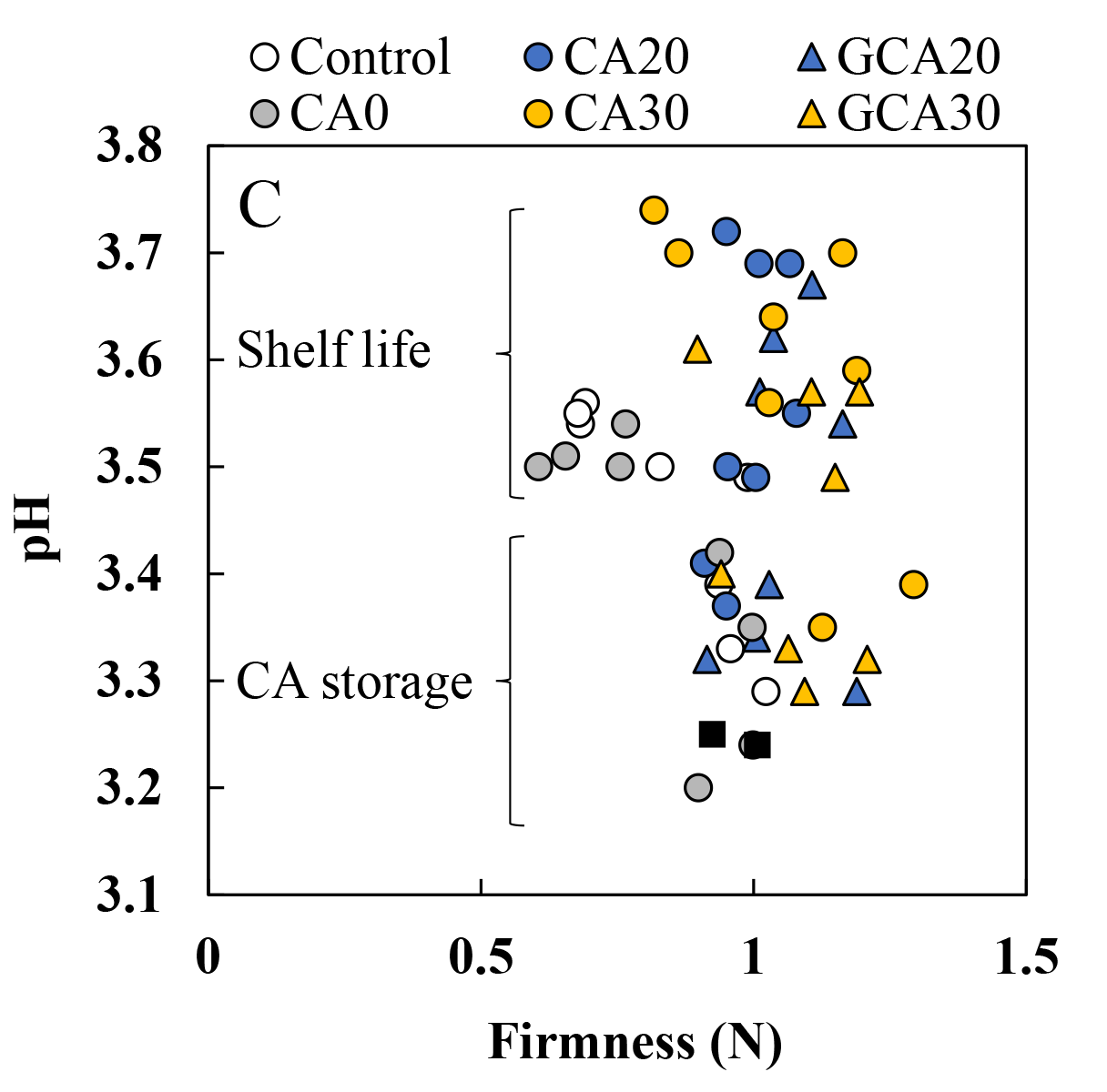

Supplement: Supplementary Figure 2 — Correlation between pH and firmness of fruit from different CA treatments during storage and subsequent shelf life. The closed black squares represent data of day 0. Data represent individual blocks. [file Image_2.TIF]
